# Supplementary material for: Near-critical spreading of droplets
Source: Nat Commun. 2022 Dec 2;13:7442. doi: 10.1038/s41467-022-35047-1 (PMC9718839; doi:10.1038/s41467-022-35047-1)
Supplement: Supplementary file 1 — Supplementary Information [file 41467_2022_35047_MOESM1_ESM.pdf]

Near-critical spreading of droplets

- Supplementary Information (SI) -

Raphael Saiseau, Christian Pedersen, Anwar Benjana,
Andreas Carlson, Ulysse Delabre, Thomas Salez and Jean-Pierre Delville

Supplementary Method 1: Near-critical micellar phases of microemulsion

Generally speaking, critical phenomena occur when a system is approaching a second-order phase transition. As density fluctuations grow beyond any molecular size and diverge at this transition, many behaviors of the chosen system are no more attached to the specific type of material but are instead governed by universal critical properties. The latter are valid within the so-called universality class associated to the symmetries of the system. For instance, the Ising ($d = 3, n = 1$) universality class, where d and n are the space and order-parameter dimensions, corresponds to uniaxial ferromagnetism, alloys, and also to liquid-gas and liquid–liquid mixture transitions for isotropic liquids [1]. Any result obtained from a system belonging to this class is fully transposable to any another system belonging to the same class. Close to the transition, the system properties vary as power laws of the distance to the critical point with universal exponents related to the universality class.

We use a near-critical micellar phase of microemulsion. In our case, it is composed of water, oil (toluene), surfactant (sodium-dodecylsulfate, SDS), and cosurfactant (n-butanol-1). The chosen critical composition is toluene: 70% wt, water: 9% wt, SDS: 4% wt, and butanol: 17% wt. As water and surfactant are in minority, as compared to 87% wt for toluene + butanol, the quaternary mixture can be considered as a binary one. It organizes at thermodynamic equilibrium as a nanosuspension of surfactant-coated water nanodroplets, the micelles, dispersed in a continuum mainly composed of toluene. As alcohol is miscible in both water and oil, it serves as co-surfactant to ensure the thermodynamic stability of the nanosuspension. As for any binary liquid mixture, a line of critical points

exists in the phase diagram, which separates the miscible state from the phase-separated state, the last one consisting in two micellar phases of different micellar concentrations. We stress that the coexistence curve is inverted as compared to the case of classical mixtures [2]. For the chosen critical composition, the micelle size, given by the amplitude factor of the correlation length of density fluctuations, is $\xi_0 = 20$ Å in the two-phase region [3]. This value is reasonably small to keep the mixture relatively transparent in the visible range (weak turbidity far from the critical point) and large enough to make easier observations of critical phenomena as compared to classical binary mixtures where ξ_0 is typically ten times smaller. Consisting in spherical micelles immersed in an oil continuum, this micellar phase of microemulsion is isotropic and belongs to the universality class $(d = 3, n = 1)$ of the Ising model [4]. The chosen composition presents a critical temperature $T_c \approx 38$ °C, above which the mixture separates in two micellar phases of different micelle concentrations $\Phi_{i=1,2}$, as indicated in the schematic phase diagram shown in Fig. 1a. Related to universality, many properties of the microemulsion present power-law behaviors in $(T - T_c)$ near the critical point. Of particular interest for near-critical droplet spreading in the two-phase region, are:

1) The micellar concentration of the two coexisting micellar phases (deduced from index of refraction measurement, [4, 5]): $\Phi_{i=1,2} = \Phi_c + b\left(\frac{T-T_c}{T_c}\right) \pm \frac{\Delta\Phi_0}{2}\left(\frac{T-T_c}{T_c}\right)^{\beta}$, with the universal exponent $\beta = 0.325$, the concentration at criticality $\Phi_c = 0.11$, the asymmetry parameter $b = 1.185$ and the coexistence amplitude $\Delta\Phi_0 = 0.275$.

2) The density of the two coexisting micellar phases (obtained from $\Delta\rho \approx \left(\frac{\partial\rho}{\partial\Phi}\right)\Delta\Phi$): $\rho_{i=1,2} = \rho_c + c\left(\frac{T-T_c}{T_c}\right) \pm \frac{\Delta\rho_0}{2}\left(\frac{T-T_c}{T_c}\right)^{\beta}$, with the same universal exponent $\beta = 0.325$, the density at criticality $\rho_c = 876.8\ \mathrm{kg/m^3}$, the asymmetry parameter $c = 230.5\ \mathrm{kg/m^3}$ and the coexistence amplitude $\Delta\rho_0 = 53.5\ \mathrm{kg/m^3}$.

3) The interfacial tension between the coexisting phases (measured from interface deformation by radiation pressure and liquid column breakup [6]): $\gamma = \gamma_0 \left(\frac{T-T_c}{T_c}\right)^{2\nu}$ with the universal exponent $\nu = 0.63$ and the amplitude $\gamma_0 = (5.0 \pm 0.2) \cdot 10^{-5}\ \mathrm{N/m}$.

4) The shear viscosity of the coexisting phases (measured empirically using a thermally-controlled Poiseuille viscometer [7]): $\eta_{i=1,2} = [1.46 - 0.014(T - 273)] \times (1 + 2.5\Phi_{i=1,2}) \cdot 10^{-3}\ \mathrm{Pa.s.}$

Supplementary Method 2: Image processing

Edge detection principle

When working with near-critical systems and approaching the critical point, the contrast in index of refraction between the two coexisting phases vanishes at the critical point while the turbidity (proportional to the susceptibility χ_T and a function of the correlation length ξ of density fluctuations) and the interface roughness (due to interfacial fluctuations) both diverge.

Consequently, the contrast decreases a lot and interfaces are made observable by slightly shifting the microscope objective out of focus to produce patterns of successive dark and light edges, the order of which depends on the microscope objective shift, either forward or backward relatively to the observed object (Supplementary Fig. 1a-c).

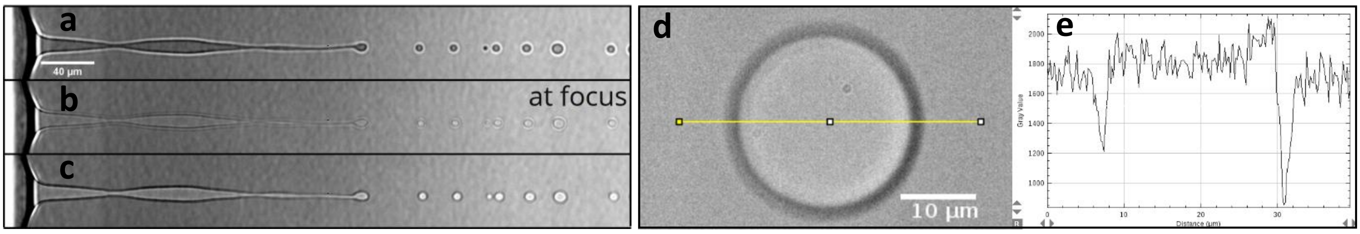

Supplementary Figure 1** – **Near-critical droplet microscopy. *Typical microscopy images of an induced liquid jet triggered on the near-critical interface of the phase-separated microemulsion* ($T - T_c = 8\ \mathrm{K}$) *with a microscope objective respectively set **a** slightly backward, **b** at focus and **c** slightly forward relatively to the observed object. **d** Microscopy image of a suspended droplet* ($T - T_c = 20\ \mathrm{K}$) *in the forward case, and **e** light intensity variations of the image section shown by the yellow line.*

This slight shift increases artificially the width of the observed interfaces on images. We display in Supplementary Fig. 1d-e an example of droplet imaging, where these variations can be appreciated when the microscope objective is slightly shifted out of focus. These variations in intensity result from the differential refraction between inside and outside the droplet. Note that the index of refraction of the droplet (composed of the micellar phase of concentration Φ_1) is larger than the one of its surrounding (composed of the micellar phase of concentration Φ_2). The droplet acts as a positive lens and thus light rays arriving from behind are bent towards its center. In the forward-shifted configuration (observation plan set behind the observed object), the dark shadows are then an effect of light depletion close to the periphery of the droplet (in an ideal configuration, the outer diameter of the shadow would correspond to the droplet diameter [8]). Moreover, using microscopy, diffraction at the interface edges is also expected to blur the external diameter. Finally, light refraction is the strongest at the edge of the droplet so that the largest light intensity variations are expected to appear close to the droplet periphery. Hence, the edge detection method considered here takes into consideration the external intensity gradient maxima at the droplet interface. In our case, focus adjustment is done to ensure that the chosen focus plane is very close to the ideal focus plane.

Edge detection algorithm

To perform the edge detection of the droplet, we use a standard algorithm based on the Canny edge detection filter [10], as described below. As explained in [8], we consider here that the droplet interface corresponds to the external intensity gradient maxima of the shadowed edge. This choice is computationally non-ambiguous, can be straightforwardly implemented algorithmically, and is robust to global intensity variations.

To obtain the external intensity gradient maxima, a three-step method is used:

- First, the raw image is divided by a background image and then filtered using a bilateral filter, chosen to be a standard Gaussian smoothing filter working preferentially on intensity variations smaller than a given threshold σ_I [9]. Here, we use a Gaussian smoothing with a characteristic size σ_x smaller than the microscope objective's lateral resolution (typically close to or below 1 µm). This smears out the electronic noise and the fluctuations in index of refraction due to the proximity to the critical point, while preserving the strong edges (see Supplementary Fig. 2b).
- Then, we use a classic Canny edge detection algorithm [10] on the filtered image. This multi-stage algorithm returns all the pixel positions corresponding to local intensity gradient maxima (calculated with a Sobel operator) based on a standard two-threshold process [10] (a low threshold ΔI_d and a high threshold ΔI_u) to get continuous lines following the image edges, even for varying gradient maxima (see Supplementary Fig. 2c).
- Lastly, we build a single contour line, using a custom-made algorithm working from the lines returned by the Canny edge detector. This algorithm is used twice to get the final contour (see Supplementary Fig. 2c).

The four parameters above (σ_I, σ_x, ΔI_d, ΔI_u) are then slightly adjusted to obtain a unique contour which correctly follows the shadow external intensity gradient maxima on every image of a given film (see Supplementary Fig. 2a).

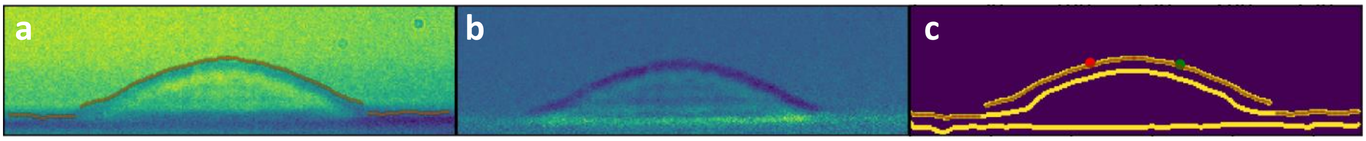

Supplementary Figure 2* – *Edge detection algorithm. ***a*** *Raw image of a spreading droplet at* $T - T_c = 1\ \mathrm{K}$*, with the final contour returned by the edge detection method.* ***b*** *Image of the same droplet when divided by the background and further filtered using a bilateral filter.* ***c*** *Canny edge detection of the same droplet in yellow. The unique contour built using the in-house algorithm is shown in orange combining contours starting from the two different initial points (in red and green).*

Extraction of contact radius R and height H of a spreading droplet

The edge detection algorithm returns a series of contours $h(r)$ (one by image) parametrized by the horizontal coordinate, which in our case corresponds to the radial coordinate *r* in the image plan ($r = 0$ corresponds to the middle of the droplet). The substrate position is visually obtained in an independent way, using the collapse of the droplet pushed by radiation pressure.

To extract the droplet radius *R* and height *H*, the contours are interpolated using a quadratic B-splines function from Scipy Python library, with a smoothing error allowed below the pixel size.

The droplet height *H* corresponds to the maximal height value of extrapolated contour, while the droplet contact radius *R* is obtained from a spherical-cap fit with three free parameters: the lateral droplet position, the maximum height and the contact radius (see Supplementary Fig. 3). Note that Bond-number corrections to sphericity [11] are negligible for the $(T - T_c)$ range investigated. The error bars associated with the measurements of *R* and *H* include: the pixel resolution and the error on the substrate position, and the errors returned by the spherical fit on both *R* and *H* (even if the *H* value is not obtained through the fit).

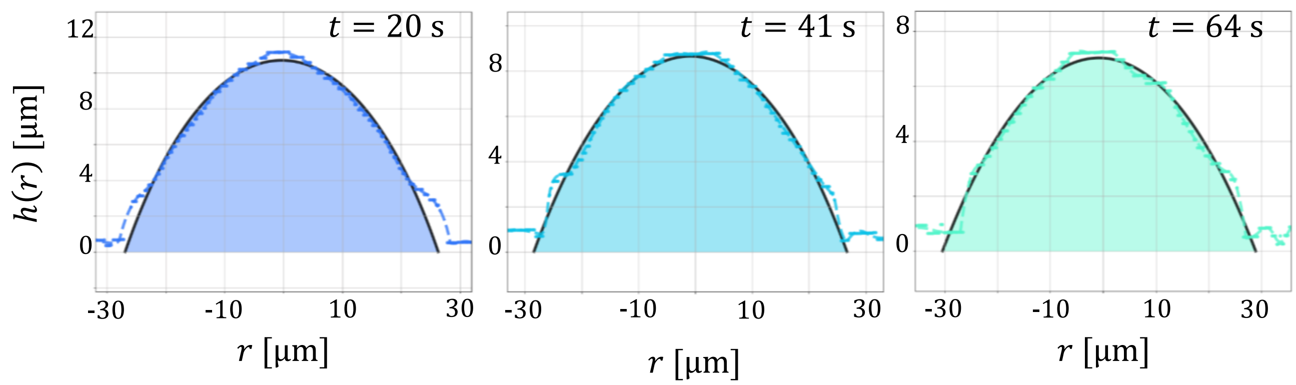

Supplementary Figure 3 – Spherical cap fits. *Droplet contour obtained using the three-step custom edge detection algorithm in dashed line and the corresponding spherical-cap fits in black solid line, for the droplet spreading at $T - T_c = 1$ K shown in Supplementary Fig. 2 at different times ($t = 0$ corresponding to the first experimental image where the curvature is homogeneous).*

Logarithmic mean procedure

The droplet height *H* and radius *R* are obtained for each image, from the droplet contour corresponding to the external gradient maxima of its shadow edge. They respectively correspond to the maximum of this contour with the substrate position subtracted and the contact radius returned by a spherical-cap fit. These quantities are obtained for droplet spreading monitored until the end of evaporation which typically lasts 2 to 6 minutes, depending on the value of $(T - T_c)$, with a frame rate varying between 50 and 300 fps. This results in data sets of 6000 to 30000 measurements for each experiment. As we are investigating power-law behaviors, this data set is reduced to several hundred points logarithmically distributed in time using a custom-made mean procedure (see Supplementary Fig. 4 for an example). The error bars on a given average radius $E_{\langle R \rangle} = \left(e_{\langle R \rangle}{}^2 + \sigma_R{}^2/N\right)^{(1/2)}$ are obtained from the standard deviation σ_R of the data distribution comprised in the time window, and the mean value $e_{\langle R \rangle}$ of the corresponding individual error distribution, with *N* being the number of points inside the time window. An example is given in Supplementary Fig. 4.

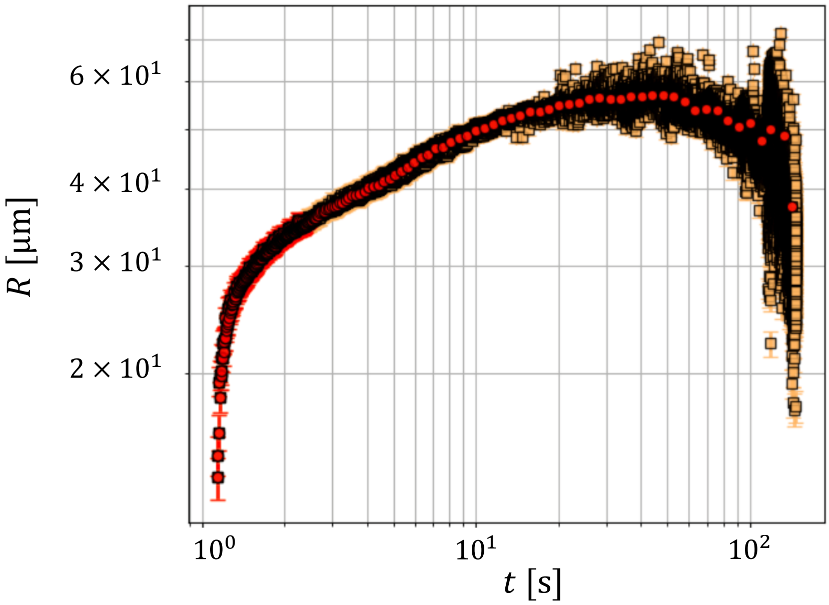

Supplementary Figure 4** – **Logarithmic mean procedure. *Contact radius R of the droplet as a function of time, obtained from fitting on the contours returned by the edge detection algorithm, for the 25600 images of a spreading droplet at $T - T_c = 8$ K (orange squared points), and the reduced data set of the 730 local mean radius obtained using the custom-made logarithmic mean procedure (red round points).*

Supplementary References

[1] Kumar, A., Krishnamurthy, H. R., & Gopal, E. S. R. Equilibrium critical phenomena in binary liquid mixtures. *Physics Reports*, **98**(2), 57-143 (1983).

[2] Meunier, J., Cazabat, A. M., Langevin, D., & Pouchelon, A. Critical behaviour in microemulsions. *J. Phys.(France) Lett*, **43**, L89-L95 (1982).

[3] Freysz, E., Laffon, E., Delville, J. P., & Ducasse, A. Phase conjugation in critical microemulsions. *Physical Review E*, **49**(3), 2141 (1994).

[4] Jean-Jean, B., Freysz, E., Ducasse, A., & Pouligny, B. Thermodiffusive and electrostrictive optical nonlinearities in critical microemulsions. *EPL (Europhysics Letters)*, **7**(3), 219 (1988).

[5] Girot, A. *Manipulation et déformations d'interfaces molles*. Ph.D. thesis, Université de Bordeaux, 2018.

[6] Girot, A., Petit, J., Saiseau, R., Guérin, T., Chraibi, H., Delabre, U., & Delville, J. P. Conical interfaces between two immiscible fluids induced by an optical laser beam. *Physical Review Letters*, **122**(17), 174501 (2019).

[7] Wunenburger, R., Issenmann, B., Brasselet, E., Loussert, C., Hourtane, V., & Delville, J. P. Fluid flows driven by light scattering. *Journal of Fluid Mechanics*, **666**, 273-307 (2011).

[8] Settles, G. S. *Schlieren and shadowgraph techniques: visualizing phenomena in transparent media* (Springer Science & Business Media, 2001).

[9] Tomasi, C., & Manduchi, R., *Bilateral filtering for gray and color images* in Sixth international conference on computer vision, Bombay, January 1998 (IEEE Cat. No. 98CH36271, 1998), p. 839-846.

[10] Canny, J. A computational approach to edge detection. *IEEE Transactions on pattern analysis and machine intelligence*, **6**, 679-698 (1986).

[11] Verma, G., Chesneau, H., Chraïbi, H., Delabre, U., Wunenburger, R., & Delville, J. P. Contactless thin-film rheology unveiled by laser-induced nanoscale interface dynamics. *Soft Matter*, **16**(34), 7904-7915 (2020).
